# Effects of SARS-COV-2 on molecules involved in vascularization and autophagy in placenta tissues

**DOI:** 10.1007/s10735-024-10228-y

**Published:** 2024-08-01

**Authors:** C. Simioni, J. M. Sanz, R. Gafà, V. Tagliatti, P. Greco, A. Passaro, L. M. Neri

**Affiliations:** 1 Department of Life Sciences and Biotechnology, University of Ferrara, Ferrara, Italy; 2 Laboratory for Technologies of Advanced Therapies (LTTA)-Electron Microscopy Center, University of Ferrara, Ferrara, Italy; 3 Department of Chemical and Pharmaceutical Sciences, University of Ferrara, Ferrara, Italy; 4 Department of Translational Medicine, University of Ferrara, Ferrara, Italy; 5 Oncohematology Department, University Hospital of Ferrara Arcispedale Sant’Anna, Ferrara, Italy; 6 Department of Medical Sciences, Obstetric and Gynecological Clinic, University of Ferrara, Ferrara, Italy

**Keywords:** SARS-CoV-2, Placenta, Immunohistochemistry, VEGF, Autophagy

## Abstract

SARS-CoV-2 infection is considered as a multi-organ disease, and several studies highlighted the relevance of the virus infection in the induction of vascular injury and tissue morphological alterations, including placenta. In this study, immunohistochemical analyses were carried out on placenta samples derived from women with COVID-19 infection at delivery (SARS-CoV-2 PCR+) or women healed from a COVID-19 infection (SARS-CoV-2 negative at delivery, SARS-CoV-2 PCR-) or women who gave birth before 2019 (Control). Angiotensin Converting Enzyme 2 (ACE2) receptor, Cluster of differentiation 147 (CD147), endothelial CD34 marker, Vascular Endothelial Growth Factor (VEGF) and total Microtubule-associated protein 1 Light Chain 3B marker (LC3B) were investigated in parallel with SPIKE protein by standard IHC. Multiplexed Immunohistochemical Consecutive Staining on Single Slide (MICSSS) was used to examine antigen co-expression in the same specimen. SPIKE protein was detected in villi and decidua from women with ongoing infection, with no significant differences in SPIKE staining between both biopsy sites. VEGF was significantly increased in SARS-CoV-2 PCR + biopsies compared to control and SARS-CoV-2 PCR- samples, and MICSSS method showed the co-localization of SPIKE with VEGF and CD34. The induction of autophagy, as suggested by the LC3B increase in SARS-CoV-2 PCR + biopsies and the co-expression of LC3B with SPIKE protein, may explain one of the different mechanisms by which placenta may react to infection. These data could provide important information on the impact that SARS-CoV-2 may have on the placenta and mother-to-fetus transmission.

**Supplementary Information:**

The online version contains supplementary material available at 10.1007/s10735-024-10228-y.

## Introduction

The RNA virus SARS-CoV-2, not identified in humans before December 2019, represents a new strain of beta Coronavirus and is responsible for COrona VIrus Disease (COVID-19) (Chams et al. 2020).

SARS-CoV-2 infection is highly heterogeneous and of varying severity. The most common symptoms of SARS-CoV-2 infection started from a common cold and fever to more serious respiratory syndromes such as pneumonia or bronchopneumonia, up to death (COVIDSurg Collaborative and GlobalSurg Collaborative 2022; Mohamadian et al. 2021; Sauter et al. 2020). Giving further evidence that SARS-CoV-2 infection has been interpreted as a multi-organ disease, this coronavirus could infect various tissues and organs, including gut, heart, kidneys and liver, or the vascular structures and male and female organs, including the placenta (Adil et al. 2021; Rakheja et al. 2022; Rizzo et al. 2021; Robba et al. 2020; Wastnedge et al. 2021; Zamboni et al. 2022).

Specifically, human placenta can be infected with SARS-CoV-2 and the virus can proliferate in placental cells (Argueta et al. 2022).

Angiotensin Converting Enzyme 2 (ACE2) receptor is widespread in tissues, is crucial for the entrance of SARS-CoV-2 in cells (Harky et al. 2023; Zaim et al. 2020) and it is expressed in the placenta (Azinheira Nobrega Cruz et al. 2021). Therefore, further attention has been paid to the study of susceptibility from SARS-CoV-2 during pregnancy (Kyle et al. 2022).

Cluster of differentiation 147 (CD147) is a transmembrane protein involved in tissue remodeling and appears to mediate macrophage activation leading to the expression of matrix metallopeptidase-9 and pro-inflammatory cytokines and chemokines (Agostinis et al. 2022; Bortolotti et al. 2021; Fenizia et al. 2021). In the placenta, CD147 is involved in the regulation of implantation, invasion and differentiation of human trophoblasts, and is reported to be involved in virus entry and considered in some literature manuscripts as a putative alternative receptor (Behl et al. 2022; Bortolotti et al. 2021). In addition, it is involved in HIV-1 infection and other viral infections (Pushkarsky et al. 2001).

The normal vascular pattern of placenta includes vasculogenesis and angiogenesis with the development of syncytial knots at the level of the villi and has been associated with the physiological expression of Vascular Endothelial Growth Factor (VEGF) but also with pathological conditions (Ahmad and Nawaz 2022; Alfaidy et al. 2020; Carmeliet 2005; Huang et al. 2021; Melincovici et al. 2018; Shchegolev et al. 2021).

It has been reported that SARS-CoV-2 infection significantly alters the vascular pattern of the placental tissue, inducing on the one hand impaired maternal vascular perfusion, and thus insufficient blood flow to the fetus (Di Girolamo et al. 2021), and on the other hand ‘intervillous thrombi’ and coagulation alterations, with increased thrombin production and increased intravascular inflammation (Schwartz et al. 2022; Wastnedge et al. 2021).

In the placenta, the autophagic process plays a key role in the early embryonic stages and promotes the development and remodeling of the endometrium (Mizushima and Levine 2010; Tsukamoto et al. 2008). Although the entire mechanism is not yet well understood, autophagy contributes to maintain the correct balance of the maternal-fetal components during normal placental development (Gong and Kim 2014). LC3, or microtubule-associated protein 1 Light Chain 3, is ubiquitously distributed in the body (Runwal et al. 2019), is expressed in the autophagosome membrane during the autophagic process and could elicit rapid degradation of mRNAs (Hwang et al. 2022), especially if it contains the sequence AAUAAA. The protein autophagosomal splice variant LC3B is involved in the autophagic response to SARS-CoV-2 infection, and it has also attracted considerable interest in in vitro studies with antiviral drug treatments (Chen and Zhang 2022; Gorshkov et al. 2020; Ivanova et al. 2023). Autophagy may have a dual role in the interaction between SARS-CoV-2 and host cells (Shan et al. 2023): on one hand, it could act as a defense mechanism, targeting and degrading viral components to limit viral replication, on the other hand, the virus may drive the autophagic process to its advantage, promoting its own survival and replication (Mao et al. 2019). During SARS-CoV-2 infection, autophagy is induced by both the innate and adaptive immune response and by Toll-like receptors (TLRs) (Carmona-Gutierrez et al. 2020). Recently, SARS-CoV-2, but not SARS-CoV, has been reported to induce autophagy and accumulation of autophagosomes. Autophagosomes appear to be central to both viral replication and virion release (Ivanova et al. 2023).

We characterized 15 tissue samples derived from placenta of women included in one of the following different conditions: (1) Pregnancy in the pre-pandemic period (Control); (2) SARS-CoV-2 positive subjects at the time of delivery (SARS-CoV-2 PCR+); (3) Subjects with infection during pregnancy but subsequent negativization at the time of delivery (SARS-CoV-2 PCR-).

Our results showed a clear influence of the viral infection on the molecules and processes studied.

## Materials and methods

### Study population

The project was designed as a case-control study with 15 pregnant women admitted at the time of delivery to the Azienda Ospedaliero-Universitaria Sant’Anna of Ferrara (Italy): 10 during 2020, 3 control pregnant women during 2019 and 2 control pregnant women during 2018.

The women were divided into 3 groups: 5 control pregnant women gave birth in the pre-pandemic period, 5 women were SARS-CoV-2 positive at the time of delivery (SARS-CoV-2 PCR+), 5 women contracted the infection during pregnancy but were negative at the time of delivery (SARS-CoV-2 PCR-). The infection was tested by molecular RT-PCR after nasal swab for the presence of SARS-CoV-2 RNA. Of note, none of the pregnant women were vaccinated against SARS-CoV-2. Clinical data were collected for each of the three cohorts.

### Ethical statement

The study was conducted in accordance with the ethical principles for medical research involving human subjects as required by the 2013 revision of the Helsinki Declaration—WMA Declaration of Helsinki—The Ethical Principles for Medical Research Involving Human Subjects. The present study was approved by the Local Ethics Committee (Comitato Etico di Area Vasta Emilia Centro della Regione Emilia-Romagna, CE-AVEC) with the reference number 122/2021/Oss/AOUFe. All participants have signed informed consent to be included in the study.

### Placenta sample collection and immunohistochemical analysis

The placenta samples were collected and sent to the Pathology Lab for optimal formalin fixation and histological analysis. One formalin-fixed paraffin-embedded block of placental parenchyma was selected from each case to perform immunohistochemical analyses. After macroscopic examination of the cut surfaces, multiple representative sections of the parenchyma were sampled for histology.

Placenta slides were deparaffined with xylene and rehydrated at decreasing ethanol concentrations. Heat antigen retrieval in Citrate Buffer Ph6 and Ph9, depending on the marker datasheet information, was performed. Slides were stained with the following antibodies, purchased from Abcam: SARS-CoV-2 SPIKE glycoprotein (Cat# ab272504, polyclonal, dilution 1:100), ACE2 (Cat# ab108252, clone EPR4435-2, dilution 1:250), CD147 (Cat# ab666, clone MEM-M6/1, dilution 1:100), CD34 (Cat# ab110643, clone EPR2999, dilution 1:250), and VEGF-A (Cat# ab1316, clone VG-1, 5 µg/ml concentration). LC3B antibody was purchased from Novus Biologicals (Cat# NB100-2220, polyclonal, dilution 1:250). Slides were counterstained with hematoxylin-eosin (H-E) and were imaged with Nikon Eclipse E100 microscope at different magnifications (10 or 20x). The tissues were scored based on number of positively stained cells/mm2 and the staining on selected areas was detected using ImageJ (64-bit Java 8) software.

### Multiplexed immunohistochemical consecutive staining on single slide (MICSSS)

MICSSS method represents a multiplex immunohistochemistry (IHC) platform based on multiple cycles of staining and scanning a single slide with an antibody panel that can include up to 10 markers (Remark et al. 2016). Each MICSSS staining cycle is identical to single IHC staining. To remove the previous antibody and staining, coverslips were removed from the slides at 56 degrees and were immersed at 50% of ethanol, subsequently in 70% ethanol + 1% hydrochloric acid (12 N) for 2 min and in 100% ethanol for 5 min. Slides were then bathed at higher percentages of ethanol. Heat-induced Epitope Retrieval for re-marking was then performed following the standard protocol. After each staining cycle, slides were scanned by the slide scanner ScanScope (Leica Biosystems), and the images were observed and saved with ScanScope Console (v9.0.0.1516) program. Consequently, the staining was reproduced in a single image, initially with QuPath v0.2.3 software and in a subsequent step with ImageJ (64-bit Java 8) software for the superposition of the individual staining. With MICSSS, in addition to the SPIKE protein, CD34, VEGF and LC3B staining was acquired.

### Staining quantification

After staining, tissue slides were observed under a light microscope in 20x, 1600 × 1200 pixel resolution at high contrast. For each slide, 4 representative areas of the villi and 4 of the decidua section were acquired using the NIS-Elements program by digitizing the images in TIFF format.

Imaging analysis was done by the ImageJ program (version bundled with 64-bit Java 8) using the IHC Profiler Plugin that allows to obtain the percentage of stained area at different intensities: very intense, moderate, mild, unmarked staining. The different percentages were used to calculate the H-score of each individual image.

### Statistical analysis

The Shapiro-Wilk test was used to identify variables with normal distribution. Variables such as demographic, clinical and hematochemical data with normal distribution were expressed as mean ± standard deviation and compared with ANOVA for independent samples. As for H-score data of markers with non-normal distribution were compared by the Mann-Whitney or Kruskal-Wallis tests to assess the statistically significant differences of the examined markers among two or three groups, respectively.

The Rho Spearman correlation test was used to analyze the correlations between the different markers by considering all biopsies together, independent of group or biopsy site (*N* = 30). Statistical analysis was performed using GraphPad software (version GraphPad.Prism.8.0.1.244.2b), and statistical significance was set to a p-value < 0.05.

## Results

### Clinical data

Study participants were divided into three groups: women negative for SARS-CoV-2 since their samples derived from pregnancy occurred before the virus spread (Controls); pregnant women declared positive at the molecular swab (RT-PCR) for SARS-CoV-2 at the time of delivery (SARS-CoV-2 PCR+); pregnant women who contracted the infection during pregnancy but were RT-PCR negative at the time of delivery (SARS-CoV-2 PCR-). The period of negativity ranged from 199 to 41 days before delivery with a mean of 88.4 ± 71.0 days.

No significant differences were reported between subjects for age, body mass index (BMI) and gestational age at delivery, as shown in the clinical data table (Table 1). The group of SARS-CoV-2 PCR- parturients included one woman with pre-pregnancy obesity. Two women, the first in SARS-CoV-2 PCR- group and the second in the control group, had type 2 diabetes.

Besides the aforementioned conditions, the enrolled women had no comorbidities or preexisting diseases such as hypertension or heart problems, nor did they have pregnancy-related diseases such as preeclampsia.

The hospitalization period for mothers with previous infection was 2.6 ± 0.9 days, shorter than that of the positive mothers at delivery who were hospitalized for 6.4 ± 1.5 days. Regarding COVID-19 complications, one woman of SARS-CoV-2 PCR- group reported fever (2 months before delivery) and respiratory symptoms; in the SARS-CoV-2 PCR + group, a patient with diabetes developed fever and dyspnea that necessitated the use of oxygen therapy.

Biochemical data showed no significant differences in D-Dimer (indicator of clotting risk) and aPTT (prothrombin partial time indicating coagulation rate) values between the groups and these parameters were within the normal range. In contrast, regarding hemoglobin and fibrinogen parameters, the two groups with previous or ongoing SARS-CoV-2 infection showed statistically significant higher levels when compared with the control group.

**Table 1:** Demographics and clinical data. Controls, samples were collected from pregnancies in pre-COVID-19 era; SARS-CoV-2 PCR+, samples collected from subjects with SARS-CoV-2 infection and PCR positive at delivery; SARS-CoV-2 PCR-, samples collected from subjects with SARS-CoV-2 infection during pregnancy but with negative PCR at delivery. BMI, body Mass Index; aPTT, activated partial Thromboplastin Time. Data are expressed as mean ± standard deviation. Values in bold are those statistically significant with p-value < 0.05

	Control	SARS-CoV-2 PCR+	SARS-CoV-2 PCR-	ANOVA *p*-value
Age (years)	28 ± 5	29 ± 2	29 ± 4	0.779
Pregravid BMI (kg/m2)	22 ± 05	25 ± 02	24 ± 05	0.470
Gestational age at delivery (weeks)	40 ± 02	40 ± 01	39 ± 02	0.783
Hemoglobin (g/dl)	10.0 ± 0.1	12.0 ± 0.1	12.0 ± 0.1	**0.005**
Fibrinogen (mg/dl)	330.3 ± 109.0	501.2 ± 63.1	502.4 ± 93.0	**0.039**
D-Dimer (mg/dl)	2.9 ± 2.5	3.0 ± 0.9	2.3 ± 1.3	0.788
aPTT (seconds)	0.9 ± 0.1	1.0 ± 0.1	0.9 ± 0.1	0.736

### Expression of SPIKE, ACE2 and CD147

**Fig. 1:**
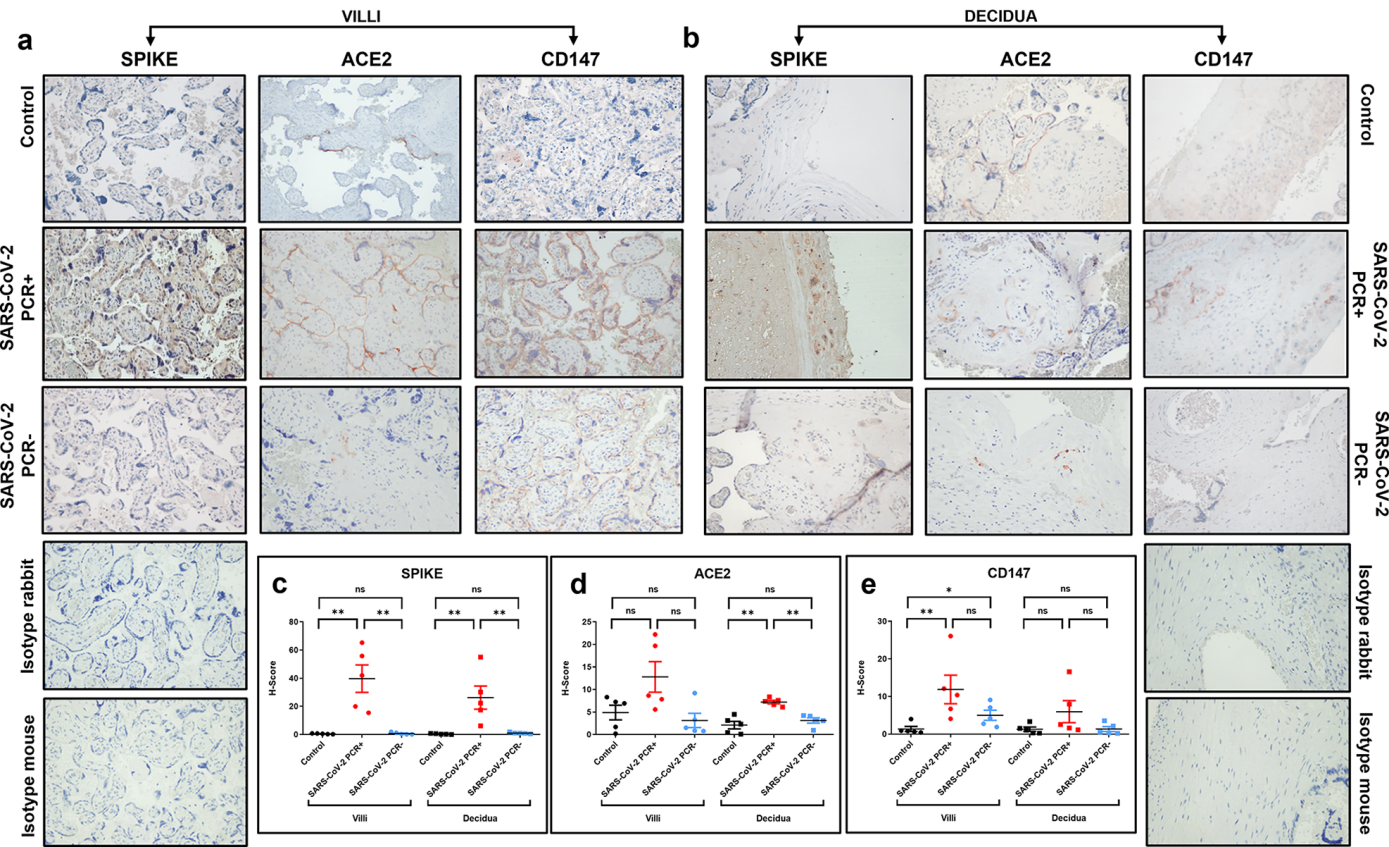
Detection of SPIKE, ACE2, and CD147 in control, SARS-CoV-2 PCR + and SARS-CoV-2 PCR- placenta. **a**: IHC images of the chorionic villi; **b**: IHC staining of the decidua. Magnifications are 20x. **c**, **d**, **e**: H-score values of SPIKE, ACE2, and CD147, respectively. p-values were obtained by Mann-Whitney test. *0.05 < p-value < 0.01; ** 0.01 < p-value < 0.001

SPIKE protein expression was detected in decidua and chorionic villi of all samples of the SARS-CoV-2 PCR + women group, whereas it was absent in the placenta of control and previously infected group (Fig. 1).

The SPIKE staining was well outlined between the villi, especially at the level of the syncytiotrophoblast (Fig. 1a). Furthermore, different areas with a more relevant or, at variance, a weaker staining were observable, therefore suggesting a staining that follows viral protein quantity. The decidua was characterized by SPIKE islets of staining, mainly located toward its external surface (Fig. 1b).

Analysis of H-score calculated from IHC images did not report differences in SPIKE staining intensity between villi and decidua in corresponding samples (Fig. 1c).

ACE2 protein was expressed in the chorionic villi, along the surface of syncytiotrophoblast for all the samples, but the expression was increased in the SARS-CoV-2 PCR + as shown in Fig. 1d.

On the contrary in the decidua, ACE2 expression was slightly higher in SARS-CoV-2 PCR + versus control and SARS-CoV-2 PCR- while no differences were detected between control and SARS-CoV-2 PCR- groups (Fig. 1b and d; Table 2).

The transmembrane protein CD147 was present at basal levels in controls and was increased in SARS-CoV-2 PCR + samples, while its expression was lower in SARS-CoV-2 PCR- placentas (Fig. 1a, b and e). In the villi, the labeling is shown tagging along the outer edge of the syncytiotrophoblast laye; while in the decidua, the staining appeared distributed inside the tissue (Fig. 1a, b).

Rho Spearman correlation coefficient was assessed by considering all the three clinical groups and the scores were obtained for each protein (SPIKE and ACE2 or CD147) at the level of the villi and decidua for each woman (Supplementary Fig. 1a, b).

The expression of SPIKE was positively associated to the presence of ACE2 in the placenta tissue (Supplementary Fig. 1a). In contrast, there was a less significant correlation between the expression of SPIKE and CD147 as shown by the Rho- and p-value (Supplementary Fig. 1b).

### SARS-CoV-2 and the expression of CD34 and VEGF in the placenta

**Fig. 2:**
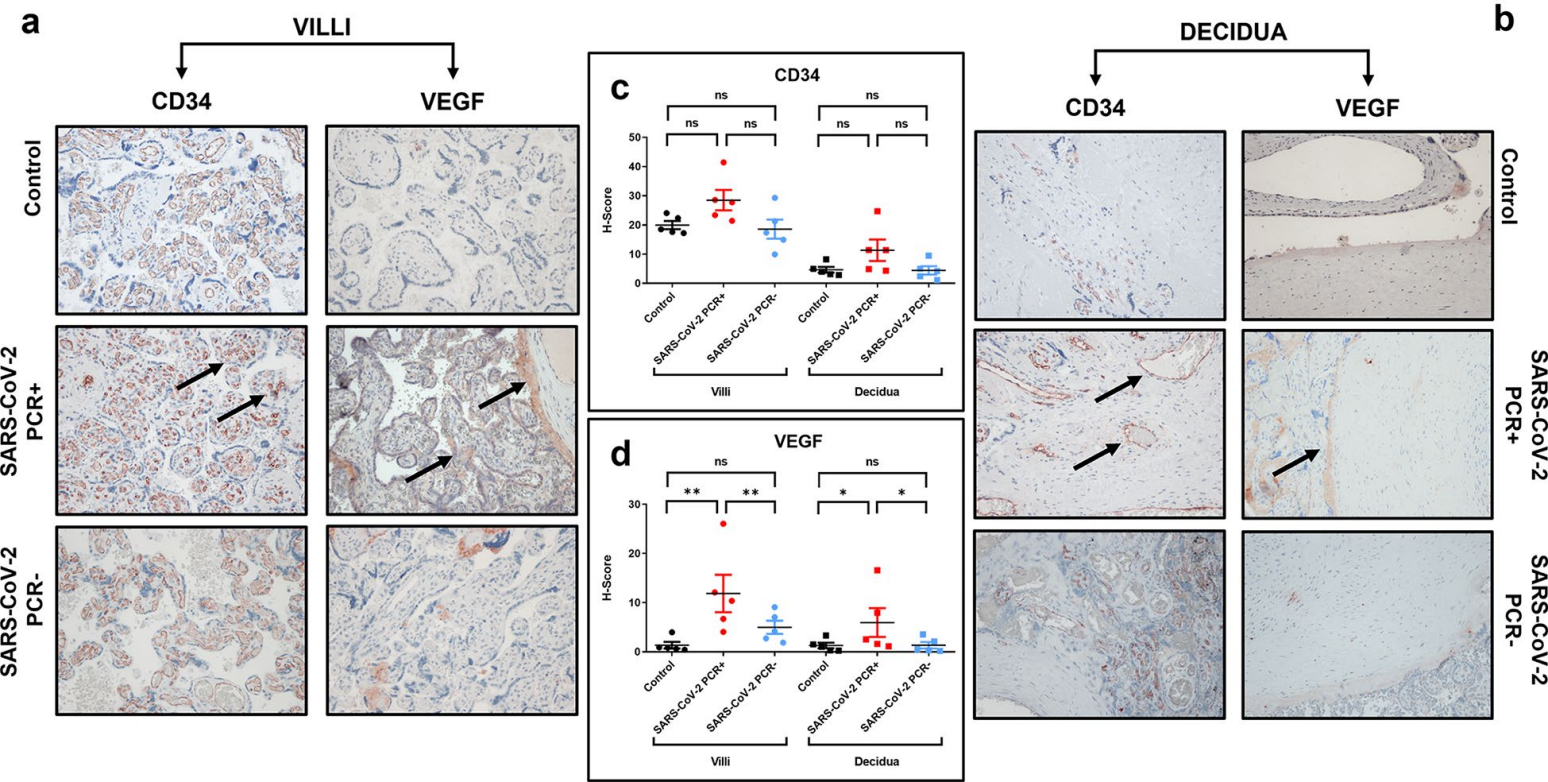
Expression of CD34 and VEGF in control, SARS-CoV-2 PCR + and SARS-CoV-2 PCR- placenta. **a**: IHC images of the chorionic villi; **b**: IHC staining of the decidua. Magnifications are 20x. **c**, **d**: H-score values of CD34 and VEGF respectively. p-values were obtained by Mann-Whitney test. ** 0.01 < p-value < 0.001. The arrows indicate the staining localization of CD34 and VEGF nearby the syncytiotrophoblast in the villi and on the surface of vascular endothelium in the decidua

IHC analysis of placenta samples with anti-VEGF and anti-CD34 antibodies was also performed. The purpose was to analyze whether SARS-CoV-2 could influence the vascular organization of the tissue, using CD34 and VEGF as readouts. IHC analysis showed that CD34 staining was localized at the level of the endothelium, nearby the syncytiotrophoblast (Fig. 2a). In the decidua, CD34 was also appreciable on the surface of vascular endothelium, near the vessels of maternal circulation (Fig. 2b). Moreover, the expression of this marker appeared more intense during SARS-CoV-2 infection (SARS-CoV-2 PCR+), even if the increase did not reach the significatively comparing with the other two groups (*p* = 0.056 *versus* control and *p* = 0.095 *versus* SARS-CoV-2 PCR- in villi and *p* = 0.095 *versus* control and *p* = 0.095 *versus* SARS-CoV-2 PCR- in decidua) (Fig. 2a, b; Table 2).

A statistically significant difference was observed for the distribution of this marker between decidua and villi with high levels in the last one (Supplementary Table 1).

Regarding VEGF, its expression was lower in control samples and SARS-CoV-2 PCR- groups when compared with SARS-CoV-2 PCR + samples, both in the villi and decidua (Fig. 2d; Table 2). In the villi, VEGF was expressed at the level of syncytiotrophoblast and in the vascular endothelium (Fig. 2a) while in the decidua the expression was most detectable in the vascular endothelium lining the blood vessels (Fig. 2b).

Rho Spearman test revealed a significant positive correlation between CD34 and VEGF (Supplementary Fig. 2a) and between SPIKE and VEGF (Supplementary Fig. 2b).

Analysis of H-score calculated from IHC images confirmed that CD34, detectable also in control samples, was more appreciable during infection, while its expression was lower in SARS-CoV-2 PCR- samples. Similarly, VEGF expression was higher during infection, while it was less appreciable in control samples and past infection (Fig. 2c, d).

**Table 2:** Analysis of the expression of the markers in the villi or decidua between control, SARS-CoV-2 PCR + and SARS-CoV-2 PCR- groups. p-value was calculated using Kruskal-Wallis or Mann-Whitney test to compare marker expression, estimated as H-score, between all the three or the two specified groups, respectively. vs.: versus. Values in bold are those statistically significant with p-value < 0.05

Marker	Biopsy site	Kruskal-Wallis *p*-value	Mann-Whitney *p*-value		
			Control vs. SARS-CoV-2 PCR+	Control vs. SARS-CoV-2 PCR-	SARS-CoV-2 PCR- vs. SARS-CoV-2 PCR+
**SPIKE**	villi	**0.009**	**0.008**	1.000	**0.008**
	decidua	**0.004**	**0.008**	0.056	**0.008**
**ACE2**	villi	0.080	0.095	0.690	0.056
	decidua	**0.008**	**0.008**	0.421	**0.008**
**CD147**	villi	**0.009**	**0.008**	**0.032**	0.151
	decidua	0.147	0.095	1.000	0.151
**CD34**	villi	0.080	0.056	0.548	0.095
	decidua	0.112	0.095	0.841	0.095
**VEGF**	villi	**0.006**	**0.008**	0.222	**0.008**
	decidua	**0.026**	**0.032**	0.841	**0.016**
**LC3B**	villi	**0.002**	**0.008**	**0.008**	**0.008**
	decidua	**0.007**	**0.008**	0.310	**0.008**

### SARS-CoV-2 induced autophagy

**Fig. 3:**
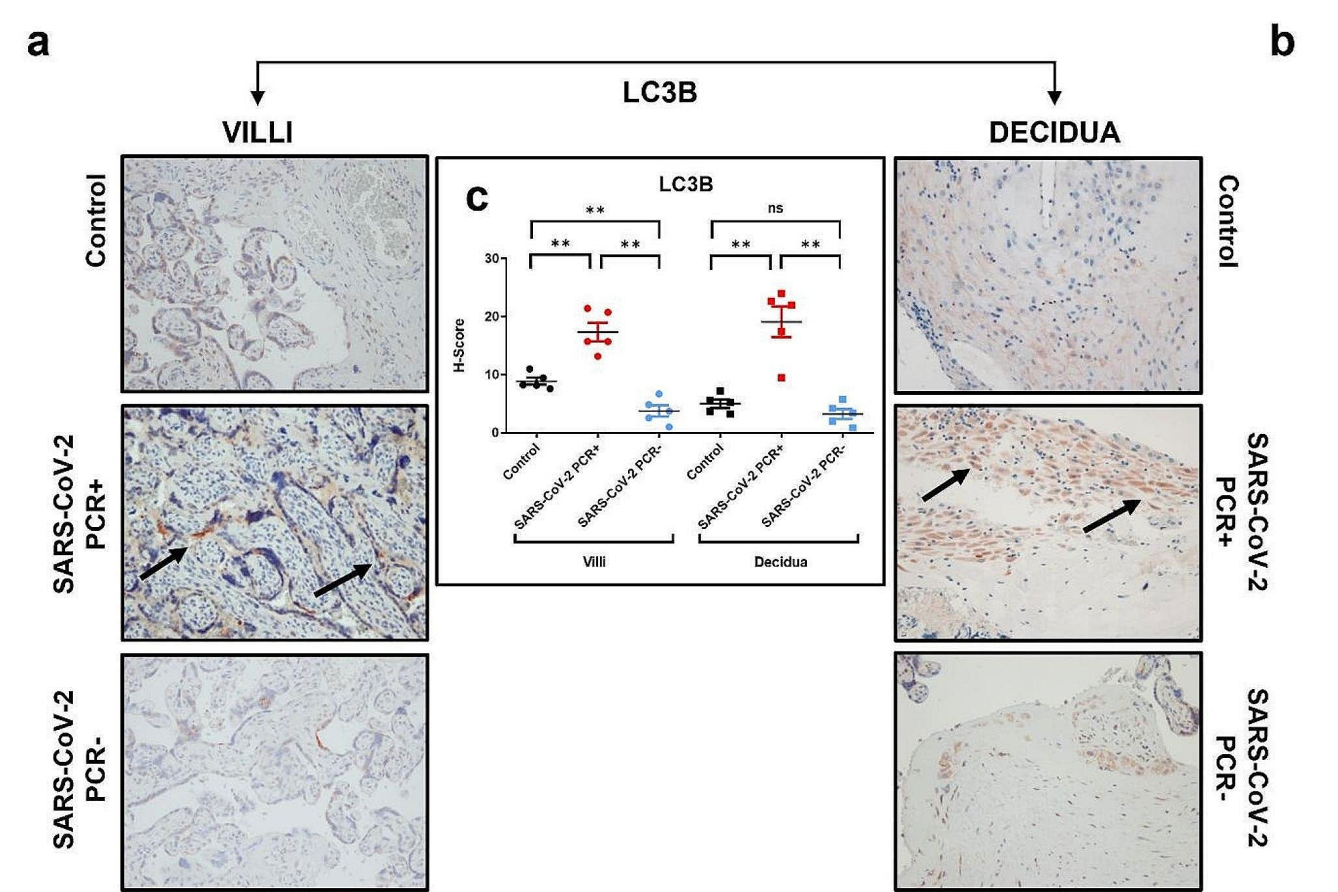
Expression of LC3B in control, SARS-CoV-2 PCR + and SARS-CoV-2 PCR- placenta. **a**: IHC images of chorionic villi; **b**: IHC staining of decidua. Magnifications are 20x. **c**: H-score values of LC3B. p-values were obtained by Mann-Whitney test. The arrows indicate the staining localization of LC3B in the villi, more widespread in the decidua

With the aim of analyzing the possible modulation of autophagy in our samples, the expression of LC3B protein was assessed. IHC analysis showed that in the normal placenta after delivery, LC3B was expressed either in the villi and in the decidua (Fig. 3a, b). In SARS-CoV-2 PCR + villi and decidua, an increase in both the spread and staining intensity was observed when compared with control and SARS-CoV-2 PCR- samples (Fig. 3; Table 2). Indeed, the H-score analysis showed a relevant increase of the PCR + samples (H-score 17–18) in comparison with the levels of control and PCR- ones. Surprisingly, LC3B expression was higher in the control than in the SARS-CoV-2 PCR- group in villi (Fig. 3; Table 2). The activation of autophagy during ongoing infection was also confirmed by the positive correlation between SPIKE and LC3B expression with the Rho Spearman correlation test (Supplementary Fig. 3).

### Analysis of tissue vascular and autophagy readouts

**Fig. 4:**
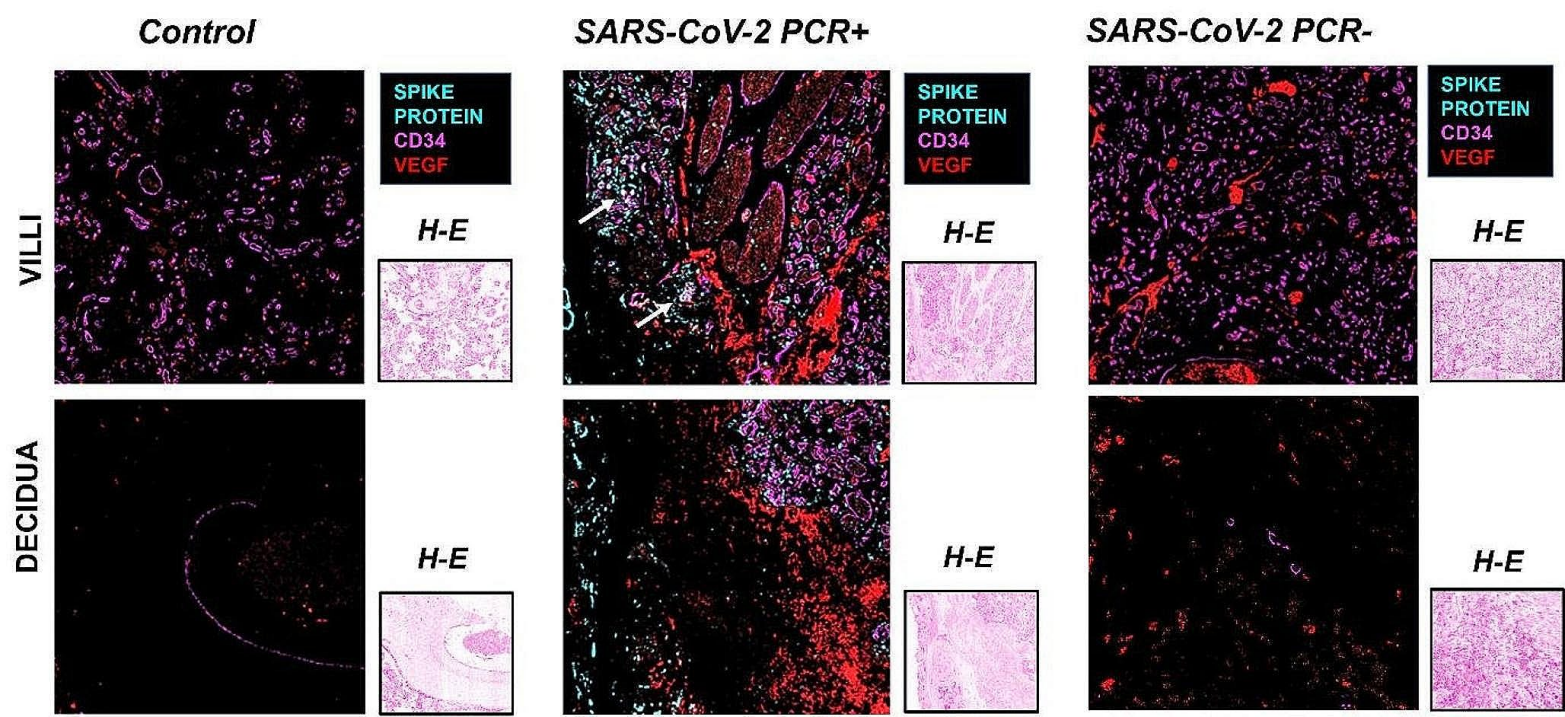
Analysis of co-expression of SPIKE, CD34 and VEGF in control, SARS-CoV-2 PCR + and SARS-CoV-2 PCR- groups in villi and decidua. The expression of the three markers SPIKE, CD34 and VEGF was co-localized. In detail, SPIKE protein was assigned the color cyan, VEGF the color red, and CD34 the color magenta. H-E: Hematoxilin Eosin. Magnifications are 20x. The arrows indicate points of co-localization of the three markers in the villi

To first analyze the co-distribution of SPIKE, CD34 and VEGF, MICSSS was performed in placenta samples at the different conditions (Fig. 4).

Similar to previous results, CD34 characterized the areas of vascular endothelium in all the three conditions, with greater expression in villi than in decidua. In particular, CD34 demarcated the capillaries in the inner part, while the more intense and thicker marking precisely indicated the surface of the vascular endothelial cells that constitute the chorionic villi. Notably, in control placenta, CD34 represented essentially the only visible staining, as there was no detection of SARS-CoV-2 and only a slight expression of VEGF. Likewise, SPIKE was not detected in the SARS-CoV-2 PCR- condition, thus indicating the absence of the virus.

The appreciable aspect of multiple staining is the co-expression, and in this case, it was most strongly expressed in the positive condition. Indeed, the strong presence of SPIKE was distributed in villi and decidua areas characterized by a high expression of VEGF and CD34 (Fig. 4).

**Fig. 5:**
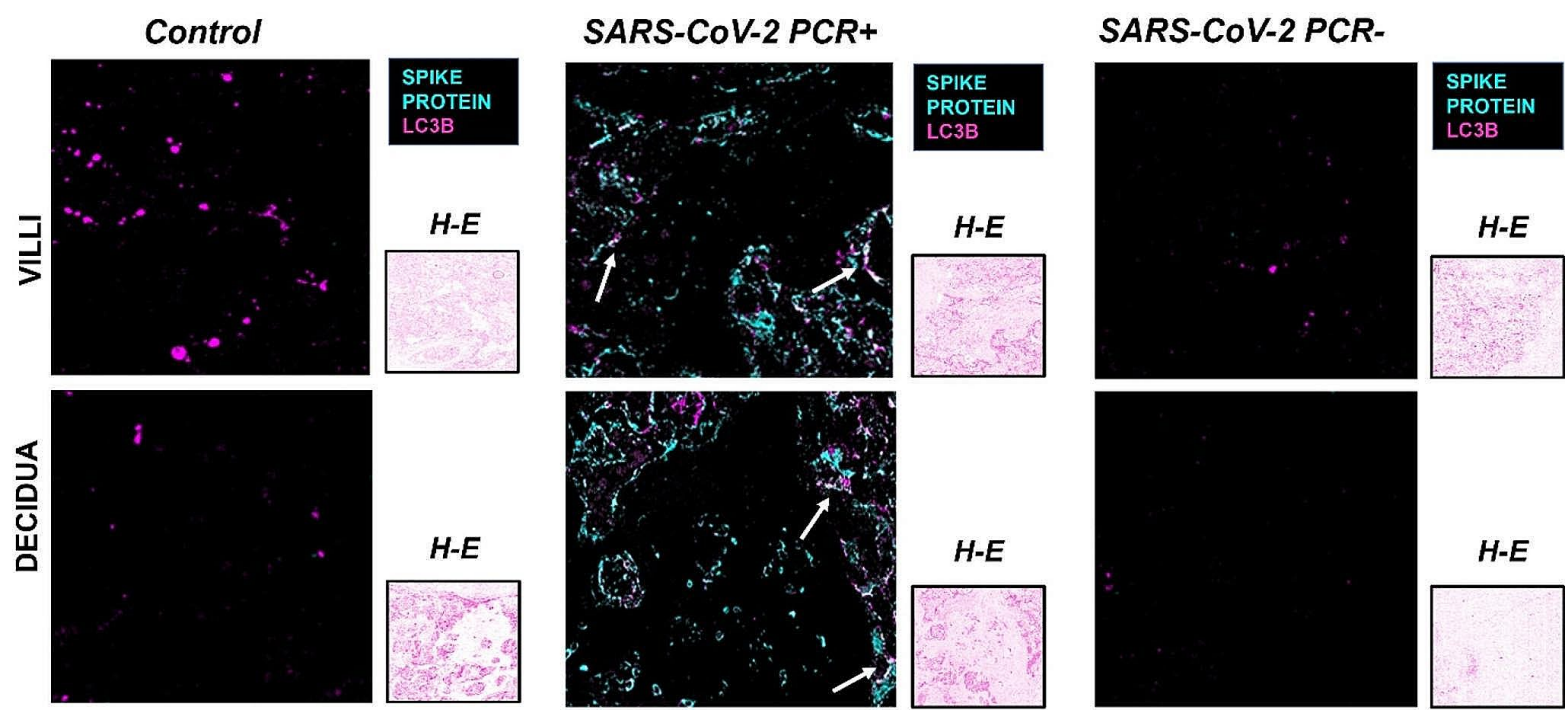
Analysis of co-expression of SPIKE and LC3B. Comparison of MICSSS of control, SARS-CoV-2 PCR + and SARS-CoV-2 PCR- conditions showing the co-localization of SPIKE and LC3B in the villi and decidua sections. With overlapping images, SPIKE protein was assigned the color cyan and LC3B the color magenta. Magnifications are 20x. The arrows indicate points of co-localization of the two markers in the villi and in the decidua

To further investigate the correlation of SARS-CoV-2 with the autophagic process, MICSSS with SPIKE protein and LC3B staining was also performed to detect whether the presence of the SPIKE protein was associated with increased autophagy (Fig. 5).

In both villi and decidua areas of SARS-CoV-2 PCR + placenta samples, the infection appeared very extensive as SPIKE staining was distributed throughout the tissue and LC3B is distributed in the proximity of the viral protein staining, both at the level of the endothelial cells that characterize the villi and in the extravillous trophoblast in the decidua (Fig. 5).

## Discussion

Since the onset of the COVID-19 pandemic, pregnant women have been particularly under observation in case of contact with SARS-CoV-2 virus to monitor the impact that its infection might have on gestation and fetus growth.

Indeed, the placenta was also considered as a possible target organ for the virus, and the scientific community has drawn attention to the possibility of vertical transmission of SARS-CoV-2 infection from mother to fetus (Moza et al. 2023; Simbar et al. 2023).

In this study, we analyzed the impact of SARS-CoV-2 infection in the modulation of markers involved in the vascular damage and the autophagic process.

The presence or the absence of the virus was assessed by the expression of the viral protein SPIKE, its main receptor ACE2 and the transmembrane protein CD147. We also analyzed the expression of VEGF, CD34 for vascular impairment and LC3B, considered as a discriminating factor in the presence or absence of autophagy.

IHC analysis confirmed the presence of SPIKE only in SARS-CoV-2 PCR + samples with staining concentrated at the level of syncytiotrophoblast and areas with staining islets in the decidua area.

ACE2 is expressed in placenta (Donoghue et al. 2000; Li et al. 2020; Valdes et al. 2006) and its expression could be increased by SARS-CoV-2 (Schiuma et al. 2023). Our data documented indeed a positive association between ACE2 and SPIKE detection.

CD147 is also present at the placental level and is involved in the placental development (Lee et al. 2013, 2022). In our samples, CD147 levels are higher in the two groups infected by SARS-CoV-2 (PCR- and PCR+) than in the control samples, suggesting that, at least in the villi, the expression is maintained even when infection is over.

SARS-CoV-2 infection can cause significant hematological and immunological alterations in maternal blood circulation that can be transmitted to the placenta. In this line, González-Mesa et al. have detected IgG anti-SARS-CoV-2 antibodies and the proinflammatory cytokines IL-1, IL-6 and IFN-γ in the umbilical cord blood of 79 pregnant women infected by SARS-CoV-2 during pregnancy (Gonzalez-Mesa et al. 2022). Inflammation is a powerful inducer of the proangiogenic marker VEGF. In fact, our results show a positive association between virus SPIKE protein and VEGF detection. These data are corroborated by the results obtained by Shchegolev et al., which showed an increase in placenta VEGF with the severity of the infection (Shchegolev et al. 2021) and increased levels of circulating VEGF has been measured in patients with worse COVID-19 prognosis (Josuttis et al. 2023; Smadja et al. 2021). Therefore, these data suggested that VEGF may represent a good predictor of adverse outcomes (Madureira and Soares 2023).

Our data showed that during SARS-CoV-2 infection, VEGF expression was increased, suggesting that this increase might be correlated to the presence of consistent alterations, disarrangements or remodeling of normal vasculature, associated with vascular endothelial injury and inflammation, presumably endothelitis. Moreover, the high expression of VEGF in SARS-CoV-2 PCR + samples was associated to the endothelial cell marker CD34, involved in various pathological conditions such as angiogenesis, or in pre-eclamptic placenta (Escudero et al. 2014). However, this aspect, which is of considerable importance, requires further investigation.

Another intriguing aspect that has been investigated was autophagy, that is a cytoplasmic process of degradation and is necessary to maintain the cellular homeostasis (Klionsky et al. 2021; Nakashima et al. 2017; Park et al. 2021; Zhao et al. 2020). Little has been studied on the role of autophagy during SARS-CoV-2 infection, especially at the placental level. Moreover, the modulation of this process in the post-infection phase is still being explored. Autophagy is a crucial process in the placenta to regulate trophoblast differentiation, endometrial development and remodeling following blastocyst implantation. Our results reported that the autophagosomal marker LC3B was expressed in healthy gestations and increased in SARS-CoV-2 PCR + placenta at the trophoblasts level but was reduced when SARS-CoV-2 infection was over (SARS-CoV-2 PCR- condition). The accumulation of LC3 in infected tissues or cells may be a mechanism exploited by SARS-CoV-2 through many cellular mechanisms to avoid the harmful effect of autophagy, as reported elsewhere (Zhou et al. 2023) and several publications also report how SARS-CoV-2 could even utilize the autophagy machinery to promote the virion production (Shan et al. 2023; Zhou et al. 2023). In our samples, an autophagic decrease in the post-infection phase could be related to the fact that the infection is overcome, and the tissue returned to a condition close to physiological. Consequently, the absence of the virus in SARS-CoV-2 PCR- placenta may prevent its own autophagosome-mediated replication with a significant decrease in LC3B marker.

It is therefore possible that autophagy could be involved in the response of placental cells to SARS-CoV-2. However, the specific implication of this process remains largely unclear and still require an in-depth analysis for different tissues, and most notably for the placenta.

It should be noted that in this study SARS-CoV-2 infection was tested, as previously detailed, at the time of delivery by a molecular swab test (RT-PCR) for the presence of SARS-CoV-2 RNA, and the placenta samples were immediately collected, fixed and subsequently analysed by means of immunohistochemistry.

The detection of the SPIKE viral glycoprotein was assumed as presence of active virus. As reported elsewhere, SPIKE was assumed as representative of SARS-CoV-2 presence in several different tissues, including lung (Bosmuller et al. 2021; Jeican et al. 2023), brain vessels (Zinserling et al. 2022), olfactory epithelium (Power Guerra et al. 2024) or liver (Swain et al. 2023).

Overall, these results resume and highlight some of the relevant modifications induced by SARS-CoV-2 infection at the placental level, that may be related with tissue and vascular damage and have effects on the mother, on the fetus and also on pregnancy.

## Electronic supplementary material

Below is the link to the electronic supplementary material.

Supplementary Material 1

## Data Availability

No datasets were generated or analysed during the current study.

## Abbreviations

ACE2: Angiotensin Converting Enzyme 2
COVID-19: Corona VIrus Disease 2019
CD147: Cluster of differentiation 147
CD34: Cluster of differentiation 34
VEGF: Vascular Endothelial Growth Factor
LC3B: Microtubule-associated protein 1 Light Chain 3B
MICSSS: Multiplexed Immunohistochemical Consecutive Staining on Single Slide
H-E: Hematoxylin-Eosin
IHC: Immunohistochemistry
IL-1: Interleukin 1
IL-6: Interleukin 6
IFN-γ: Interferon-gamma

## References

[CR1] Adil MT et al (2021) SARS-CoV-2 and the pandemic of COVID-19. Postgrad Med J 97:110–116. 10.1136/postgradmedj-2020-138386 32788312 10.1136/postgradmedj-2020-138386 PMC10016996

[CR2] Agostinis C et al (2022) SARS-CoV-2 modulates virus receptor expression in placenta and can induce trophoblast fusion, inflammation and endothelial permeability. Front Immunol 13:957224. 10.3389/fimmu.2022.957224 36177036 10.3389/fimmu.2022.957224 PMC9513489

[CR3] Ahmad A, Nawaz MI (2022) Molecular mechanism of VEGF and its role in pathological angiogenesis. J Cell Biochem 123:1938–1965. 10.1002/jcb.30344 36288574 10.1002/jcb.30344

[CR4] Alfaidy N et al (2020) The emerging role of the prokineticins and Homeobox Genes in the vascularization of the placenta: physiological and pathological aspects. Front Physiol 11:591850. 10.3389/fphys.2020.591850 33281622 10.3389/fphys.2020.591850 PMC7689260

[CR5] Argueta LB et al (2022) Inflammatory responses in the placenta upon SARS-CoV-2 infection late in pregnancy. iScience 25:104223. 10.1016/j.isci.2022.104223 35434541 10.1016/j.isci.2022.104223 PMC8996470

[CR6] Azinheira Nobrega Cruz N, Stoll D, Casarini DE, Bertagnolli M (2021) Role of ACE2 in pregnancy and potential implications for COVID-19 susceptibility. Clin Sci (Lond) 135:1805–1824. 10.1042/CS20210284 34338772 10.1042/CS20210284 PMC8329853

[CR7] Behl T et al (2022) CD147-spike protein interaction in COVID-19: get the ball rolling with a novel receptor and therapeutic target. Sci Total Environ 808:152072. 10.1016/j.scitotenv.2021.152072 34863742 10.1016/j.scitotenv.2021.152072 PMC8634688

[CR8] Bortolotti D et al (2021) Relevance of VEGF and CD147 in different SARS-CoV-2 positive digestive tracts characterized by thrombotic damage. FASEB J 35:e21969. 10.1096/fj.202100821RRR 34822202 10.1096/fj.202100821RRR

[CR9] Bosmuller H, Matter M, Fend F, Tzankov A (2021) The pulmonary pathology of COVID-19. Virchows Arch 478:137–150. 10.1007/s00428-021-03053-1 33604758 10.1007/s00428-021-03053-1 PMC7892326

[CR10] Carmeliet P (2005) VEGF as a key mediator of angiogenesis in cancer. Oncol 69 Suppl 3:4–10. 10.1159/000088478 10.1159/000088478 16301830

[CR11] Carmona-Gutierrez D, Bauer MA, Zimmermann A, Kainz K, Hofer SJ, Kroemer G, Madeo F (2020) Digesting the crisis: autophagy and coronaviruses. Microb Cell 7:119–128. 10.15698/mic2020.05.715 32391393 10.15698/mic2020.05.715 PMC7199282

[CR12] Chams N et al (2020) COVID-19: a multidisciplinary review. Front Public Health 8:383. 10.3389/fpubh.2020.00383 32850602 10.3389/fpubh.2020.00383 PMC7403483

[CR13] Chen D, Zhang H (2022) Autophagy in severe acute respiratory syndrome coronavirus 2 infection. Curr Opin Physiol 29:100596. 10.1016/j.cophys.2022.100596 36187896 10.1016/j.cophys.2022.100596 PMC9514017

[CR14] COVIDSurg Collaborative, GlobalSurg Collaborative (2022) SARS-CoV-2 infection and venous thromboembolism after surgery: an international prospective cohort study. Anaesthesia 77:28–39. 10.1111/anae.15563 34428858 10.1111/anae.15563 PMC8652887

[CR15] Di Girolamo R et al (2021) Placental histopathology after SARS-CoV-2 infection in pregnancy: a systematic review and meta-analysis. Am J Obstet Gynecol MFM 3:100468. 10.1016/j.ajogmf.2021.100468 34425296 10.1016/j.ajogmf.2021.100468 PMC8379009

[CR16] Donoghue M et al (2000) A novel angiotensin-converting enzyme-related carboxypeptidase (ACE2) converts angiotensin I to angiotensin 1–9. Circ Res 87:E1–9. 10.1161/01.res.87.5.e1 10969042 10.1161/01.res.87.5.e1

[CR17] Escudero C et al (2014) Increased placental angiogenesis in late and early onset pre-eclampsia is associated with differential activation of vascular endothelial growth factor receptor 2. Placenta 35:207–215. 10.1016/j.placenta.2014.01.007 24508097 10.1016/j.placenta.2014.01.007

[CR18] Fenizia C, Galbiati S, Vanetti C, Vago R, Clerici M, Tacchetti C, Daniele T (2021) SARS-CoV-2 entry: at the crossroads of CD147 and ACE2. Cells 10. 10.3390/cells10061434 10.3390/cells10061434 PMC8226513 34201214

[CR19] Gong JS, Kim GJ (2014) The role of autophagy in the placenta as a regulator of cell death. Clin Exp Reprod Med 41:97–107. 10.5653/cerm.2014.41.3.97 25309853 10.5653/cerm.2014.41.3.97 PMC4192457

[CR20] Gonzalez-Mesa E et al (2022) Transmitted fetal Immune response in cases of SARS-CoV-2 infections during pregnancy. Diagnostics (Basel) 12. 10.3390/diagnostics12020245 10.3390/diagnostics12020245 PMC8870756 35204335

[CR21] Gorshkov K et al (2020) The SARS-CoV-2 cytopathic effect is blocked with autophagy modulators. bioRxiv. 10.1101/2020.05.16.091520

[CR22] Harky A, Ala’Aldeen A, Butt S, Duric B, Roy S, Zeinah M (2023) COVID-19 and Multiorgan Response: the long-term impact. Curr Probl Cardiol 48:101756. 10.1016/j.cpcardiol.2023.101756 37088175 10.1016/j.cpcardiol.2023.101756 PMC10122551

[CR23] Huang Z, Huang S, Song T, Yin Y, Tan C (2021) Placental angiogenesis in mammals: a review of the Regulatory effects of Signaling pathways and functional nutrients. Adv Nutr 12:2415–2434. 10.1093/advances/nmab070 34167152 10.1093/advances/nmab070 PMC8634476

[CR24] Hwang HJ, Ha H, Lee BS, Kim BH, Song HK, Kim YK (2022) LC3B is an RNA-binding protein to trigger rapid mRNA degradation during autophagy. Nat Commun 13:1436. 10.1038/s41467-022-29139-1 35302060 10.1038/s41467-022-29139-1 PMC8931120

[CR25] Ivanova T et al (2023) Autophagy and SARS-CoV-2-Old players in New games. Int J Mol Sci 24. 10.3390/ijms24097734 10.3390/ijms24097734 PMC10178552 37175443

[CR26] Jeican II et al (2023) Histopathological lung findings in COVID-19 B.1.617.2 SARS-CoV-2 Delta variant. J Pers Med 13. 10.3390/jpm13020279 10.3390/jpm13020279 PMC9961426 36836513

[CR27] Josuttis D, Schwedler C, Heymann G, Gumbel D, Schmittner MD, Kruse M, Hoppe B (2023) Vascular endothelial growth factor as potential biomarker for COVID-19 severity. J Intensive Care Med 38:1165–1173. 10.1177/08850666231186787 37448220 10.1177/08850666231186787 PMC10345830

[CR28] Klionsky DJ et al (2021) Guidelines for the use and interpretation of assays for monitoring autophagy (4th edition)(1). Autophagy 17:1–382. 10.1080/15548627.2020.1797280 33634751 10.1080/15548627.2020.1797280 PMC7996087

[CR29] Kyle MH, Hussain M, Saltz V, Mollicone I, Bence M, Dumitriu D (2022) Vertical Transmission and neonatal outcomes following maternal SARS-CoV-2 infection during pregnancy. Clin Obstet Gynecol 65:195–202. 10.1097/GRF.0000000000000667 35045041 10.1097/GRF.0000000000000667 PMC8767921

[CR31] Lee CL et al (2013) Identification of CD147 (basigin) as a mediator of trophoblast functions. Hum Reprod 28:2920–2929. 10.1093/humrep/det355 24014600 10.1093/humrep/det355

[CR30] Lee CL et al (2022) Dysregulation of the CD147 complex confers defective placental development: a pathogenesis of early-onset preeclampsia. Clin Transl Med 12:e826. 10.1002/ctm2.826 35653421 10.1002/ctm2.826 PMC9162301

[CR32] Li M, Chen L, Zhang J, Xiong C, Li X (2020) The SARS-CoV-2 receptor ACE2 expression of maternal-fetal interface and fetal organs by single-cell transcriptome study. PLoS ONE 15:e0230295. 10.1371/journal.pone.0230295 32298273 10.1371/journal.pone.0230295 PMC7161957

[CR33] Madureira G, Soares R (2023) The misunderstood link between SARS-CoV-2 and angiogenesis. A narrative review. Pulmonology 29:323–331. 10.1016/j.pulmoe.2021.08.004 34593362 10.1016/j.pulmoe.2021.08.004 PMC8390375

[CR34] Mao J, Lin E, He L, Yu J, Tan P, Zhou Y (2019) Autophagy and viral infection. Adv Exp Med Biol 1209:55–78. 10.1007/978-981-15-0606-2_5 31728865 10.1007/978-981-15-0606-2_5 PMC7122562

[CR35] Melincovici CS et al (2018) Vascular endothelial growth factor (VEGF) - key factor in normal and pathological angiogenesis. Rom J Morphol Embryol 59:455–467 30173249

[CR36] Mizushima N, Levine B (2010) Autophagy in mammalian development and differentiation. Nat Cell Biol 12:823–830. 10.1038/ncb0910-823 20811354 10.1038/ncb0910-823 PMC3127249

[CR37] Mohamadian M, Chiti H, Shoghli A, Biglari S, Parsamanesh N, Esmaeilzadeh A (2021) COVID-19: Virology, biology and novel laboratory diagnosis. J Gene Med 23:e3303. 10.1002/jgm.3303 33305456 10.1002/jgm.3303 PMC7883242

[CR38] Moza A et al (2023) Outcome of newborns with confirmed or possible SARS-CoV-2 Vertical Infection-A scoping review. Diagnostics (Basel) 13. 10.3390/diagnostics13020245 10.3390/diagnostics13020245 PMC9858608 36673058

[CR39] Nakashima A et al (2017) Role of autophagy in oocytogenesis, embryogenesis, implantation, and pathophysiology of pre-eclampsia. J Obstet Gynaecol Res 43:633–643. 10.1111/jog.13292 28418212 10.1111/jog.13292

[CR40] Park H, Cho M, Do Y, Park JK, Bae SJ, Joo J, Ha KT (2021) Autophagy as a therapeutic target of Natural products enhancing embryo implantation. Pharmaceuticals (Basel) 15. 10.3390/ph15010053 10.3390/ph15010053 PMC8779555 35056110

[CR41] Power Guerra N, Bierkamper M, Pablik J, Hummel T, Witt M (2024) Histochemical Evidence for Reduced Immune Response in nasal mucosa of patients with COVID-19. Int J Mol Sci 25. 10.3390/ijms25084427 10.3390/ijms25084427 PMC11050322 38674011

[CR42] Pushkarsky T et al (2001) CD147 facilitates HIV-1 infection by interacting with virus-associated cyclophilin A. Proc Natl Acad Sci U S A 98:6360–6365. 10.1073/pnas.111583198 11353871 10.1073/pnas.111583198 PMC33473

[CR43] Rakheja D et al (2022) SARS-CoV-2 immunohistochemistry in Placenta. Int J Surg Pathol 30:393–396. 10.1177/10668969211067754 34939436 10.1177/10668969211067754 PMC9111943

[CR44] Remark R et al (2016) In-depth tissue profiling using multiplexed immunohistochemical consecutive staining on single slide. Sci Immunol 1:aaf6925. 10.1126/sciimmunol.aaf6925 28783673 10.1126/sciimmunol.aaf6925 PMC10152404

[CR45] Rizzo R et al (2021) SARS-CoV-2 nucleocapsid protein and ultrastructural modifications in small bowel of a 4-week-negative COVID-19 patient. Clin Microbiol Infect 27:936–937. 10.1016/j.cmi.2021.01.012 33465499 10.1016/j.cmi.2021.01.012 PMC7832059

[CR46] Robba C, Battaglini D, Pelosi P, Rocco PRM (2020) Multiple organ dysfunction in SARS-CoV-2: MODS-CoV-2. Expert Rev Respir Med 14:865–868. 10.1080/17476348.2020.1778470 32567404 10.1080/17476348.2020.1778470 PMC7441756

[CR47] Runwal G, Stamatakou E, Siddiqi FH, Puri C, Zhu Y, Rubinsztein DC (2019) LC3-positive structures are prominent in autophagy-deficient cells. Sci Rep 9:10147. 10.1038/s41598-019-46657-z 31300716 10.1038/s41598-019-46657-z PMC6625982

[CR48] Sauter JL et al (2020) Insights into pathogenesis of fatal COVID-19 pneumonia from histopathology with immunohistochemical and viral RNA studies. Histopathology 77:915–925. 10.1111/his.14201 32614086 10.1111/his.14201 PMC7361244

[CR49] Schiuma G et al (2023) Effect of SARS-CoV-2 infection in pregnancy on CD147, ACE2 and HLA-G expression. Placenta 132:38–43. 10.1016/j.placenta.2023.01.004 36628848 10.1016/j.placenta.2023.01.004 PMC9814282

[CR50] Schwartz DA et al (2022) Placental tissue Destruction and Insufficiency from COVID-19 causes stillbirth and neonatal death from hypoxic-ischemic Injury. Arch Pathol Lab Med 146:660–676. 10.5858/arpa.2022-0029-SA 35142798 10.5858/arpa.2022-0029-SA

[CR51] Shan T, Li LY, Yang JM, Cheng Y (2023) Role and clinical implication of autophagy in COVID-19. Virol J 20:125. 10.1186/s12985-023-02069-0 37328875 10.1186/s12985-023-02069-0 PMC10276507

[CR52] Shchegolev AI, Kulikova GV, Lyapin VM, Shmakov RG, Sukhikh GT (2021) The number of Syncytial knots and VEGF expression in Placental Villi in Parturient Woman with COVID-19 depends on the Disease Severity. Bull Exp Biol Med 171:399–403. 10.1007/s10517-021-05236-x 34292445 10.1007/s10517-021-05236-x PMC8295457

[CR53] Simbar M, Nazarpour S, Sheidaei A (2023) Evaluation of pregnancy outcomes in mothers with COVID-19 infection: a systematic review and meta-analysis. J Obstet Gynaecol 43:2162867. 10.1080/01443615.2022.2162867 36651606 10.1080/01443615.2022.2162867

[CR54] Smadja DM et al (2021) Placental growth factor level in plasma predicts COVID-19 severity and in-hospital mortality. J Thromb Haemost 19:1823–1830. 10.1111/jth.15339 33830623 10.1111/jth.15339 PMC8250221

[CR55] Swain LA, Ambasta A, Munhoz EP, Omodon O, Urbanski SJ, Nguyen HH (2023) Acute severe hepatitis as a presenting symptom in clinically stable patients admitted with SARS-CoV-2 Omicron infection. Hepatol Commun 7. 10.1097/HC9.0000000000000115 10.1097/HC9.0000000000000115 PMC10069854 36996001

[CR56] Tsukamoto S, Kuma A, Mizushima N (2008) The role of autophagy during the oocyte-to-embryo transition. Autophagy 4:1076–1078. 10.4161/auto.7065 18849666 10.4161/auto.7065

[CR57] Valdes G et al (2006) Distribution of angiotensin-(1–7) and ACE2 in human placentas of normal and pathological pregnancies. Placenta 27:200–207. 10.1016/j.placenta.2005.02.015 16338465 10.1016/j.placenta.2005.02.015

[CR58] Wastnedge EAN, Reynolds RM, van Boeckel SR, Stock SJ, Denison FC, Maybin JA, Critchley HOD (2021) Pregnancy and COVID-19. Physiol Rev 101:303–318. 10.1152/physrev.00024.2020 32969772 10.1152/physrev.00024.2020 PMC7686875

[CR59] Zaim S, Chong JH, Sankaranarayanan V, Harky A (2020) COVID-19 and Multiorgan Response. Curr Probl Cardiol 45:100618. 10.1016/j.cpcardiol.2020.100618 32439197 10.1016/j.cpcardiol.2020.100618 PMC7187881

[CR60] Zamboni P et al (2022) Bowel ischemia as onset of COVID-19 in otherwise asymptomatic patients with persistently negative swab. J Intern Med 291:224–231. 10.1111/joim.13385 34437741 10.1111/joim.13385 PMC8662187

[CR61] Zhao X, Jiang Y, Jiang T, Han X, Wang Y, Chen L, Feng X (2020) Physiological and pathological regulation of autophagy in pregnancy. Arch Gynecol Obstet 302:293–303. 10.1007/s00404-020-05607-1 32556514 10.1007/s00404-020-05607-1

[CR62] Zhou H, Hu Z, Castro-Gonzalez S (2023) Bidirectional interplay between SARS-CoV-2 and autophagy. mBio 14:e0102023. 10.1128/mbio.01020-23 37436071 10.1128/mbio.01020-23 PMC10470609

[CR63] Zinserling VA, Semenova NY, Bikmurzina AE, Kruglova NM, Rybalchenko OV, Markov AG (2022) SARS-CoV-2-Induced Pathology-Relevance to COVID-19 pathophysiology. Pathophysiology 29:281–297. 10.3390/pathophysiology29020021 35736649 10.3390/pathophysiology29020021 PMC9229620

