# Supplementary material for: Effects of SARS-COV-2 on molecules involved in vascularization and autophagy in placenta tissues
Source: J Mol Histol. 2024 Aug 1;55(5):753–64. doi: 10.1007/s10735-024-10228-y (PMC11464539; doi:10.1007/s10735-024-10228-y)
Supplement: Supplementary file 1 — Supplementary Material 1 [file 10735_2024_10228_MOESM1_ESM.docx]

**Supplementary Table and Figures**

|  | **All**  N=15 | **Control**  N=5 | **SARS-CoV-2 PCR+**  N=5 | **SARS-CoV-2 PCR-**  N=5 |
| --- | --- | --- | --- | --- |
|  | p-value | p-value | p-value | p-value |
| **SPIKE** | 0.902 | 0.841 | 0.421 | 0.548 |
| **ACE2** | 0.285 | 0.31 | 0.222 | 0.421 |
| **CD147** | 0.045 | 1 | 0.222 | 0.056 |
| **VEGF** | 0.461 | 0.69 | 1 | 0.222 |
| **CD34** | **<0.001** | **0.008** | **0.032** | **0.008** |
| **LC3B** | 0.461 | **0.008** | 0.31 | 0.69 |

**Table S1.** **Analysis of the expression of the markers between the villi or decidua**. p-value was calculated using Mann-Whitney test to compare marker expression, estimated as H-score, between the villi and decidua. Values in bold are those statistically significant with p-value <0.05


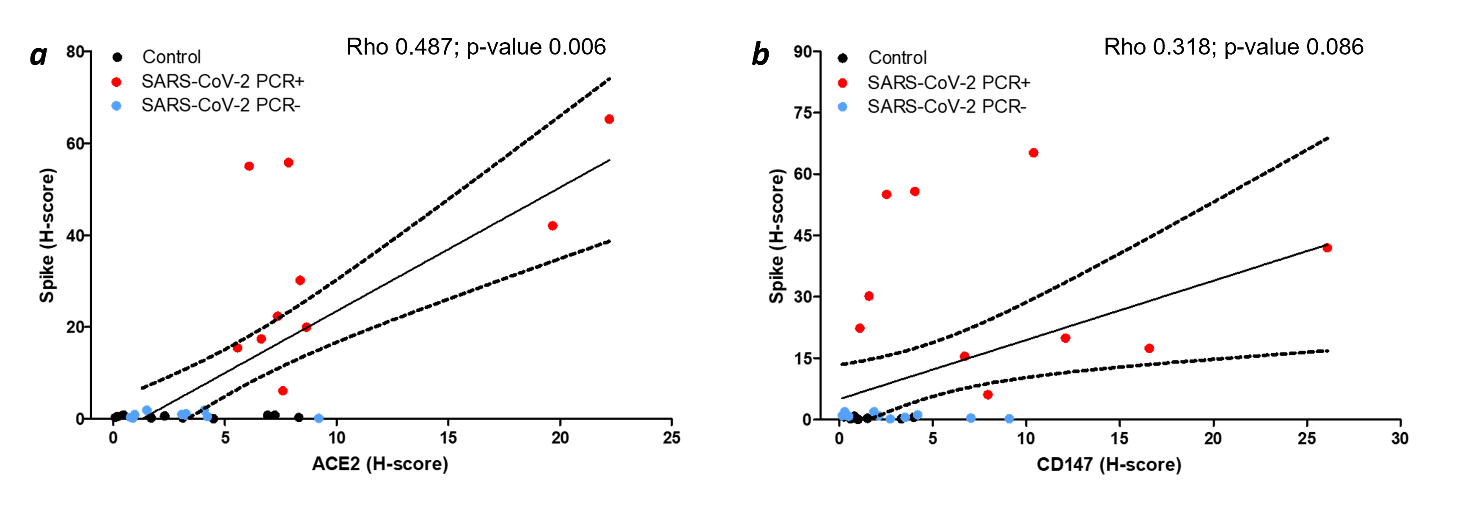


**Supplementary Fig. 1** Correlation analysis. **a:** Rho Spearman correlation between SPIKE and ACE2 expression estimated by H-score; **b:** Rho Spearman correlation between SPIKE and CD147 expression estimated by H-score Rho: Rho Spearman coefficient


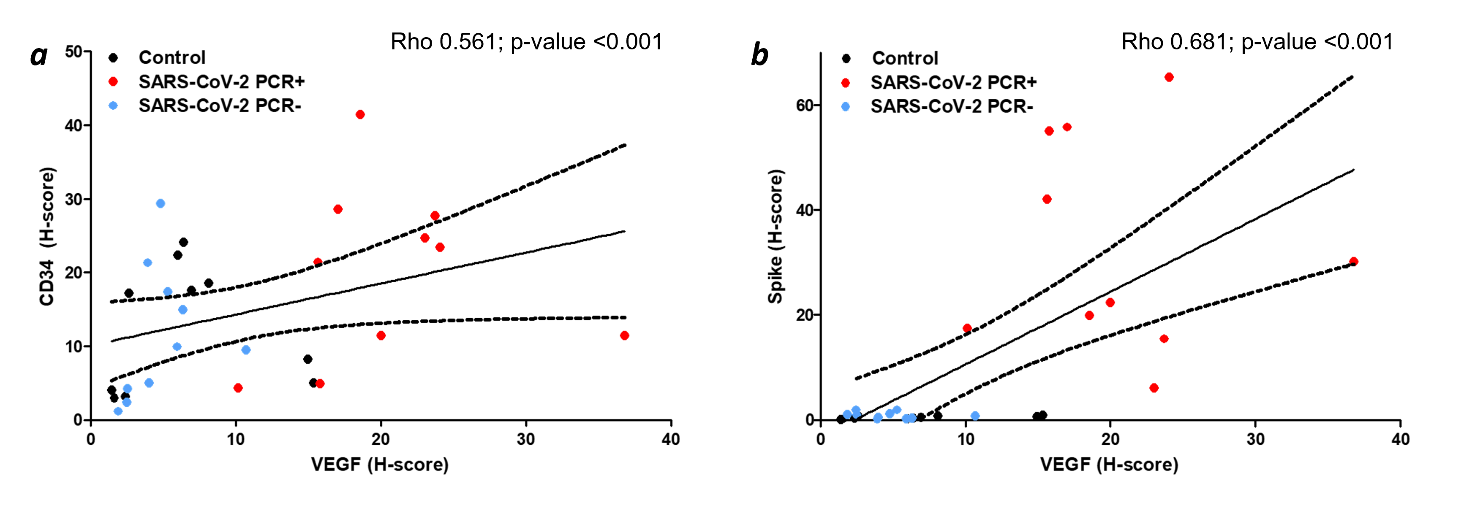


**Supplementary Fig.2** Correlation analysis. **a:** Rho Spearman correlation between CD34 and VEGF expression estimated by H-score; **b:** Rho Spearman correlation between SPIKE and VEGF expression estimated by H-score. Rho: Rho Spearman coefficient


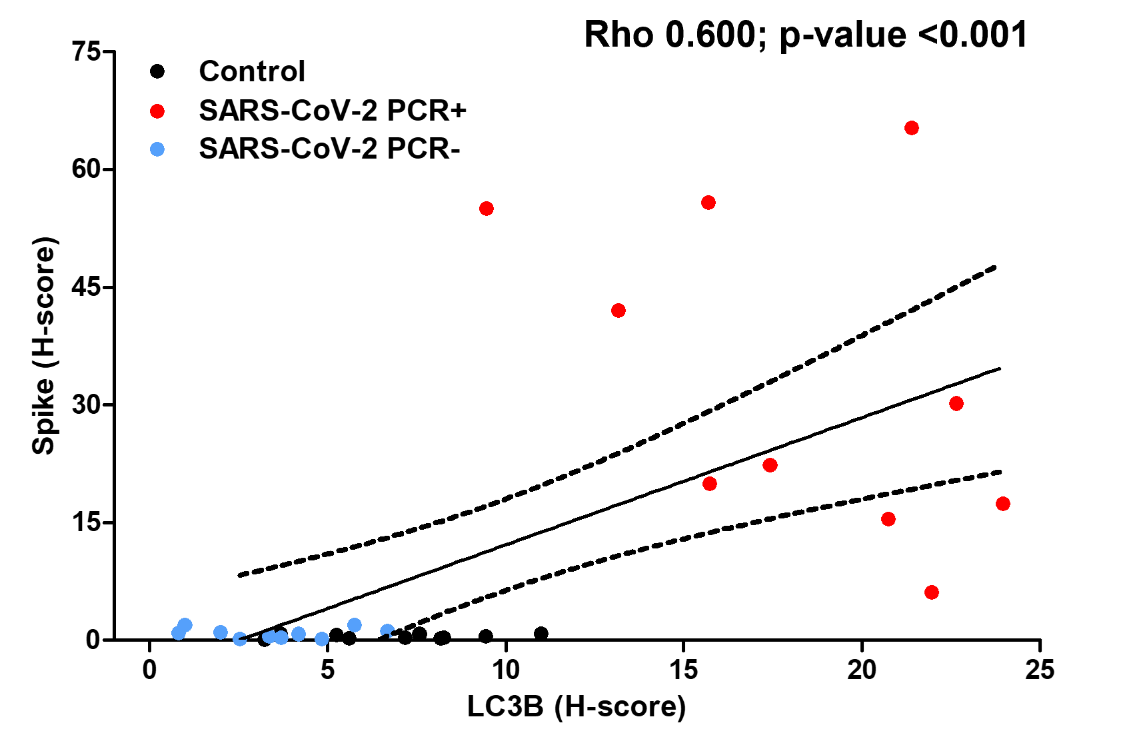


**Supplementary Fig. 3** Spearman correlation analysis between SPIKE and LC3B expression estimated by H-score. Rho: Rho Spearman coefficient
